# Congenital Cholesteatoma Localized to the Mastoid Cavity and Presenting as a Mastoid Abscess

**DOI:** 10.1155/2015/305494

**Published:** 2015-04-15

**Authors:** Salim M. Sloma Tabook, Hazem M. Abdel Tawab, Naveen Kumar Gopal

**Affiliations:** ^1^Department of Otorhinolaryngology, Sultan Qaboos Hospital, Salalah, Oman; ^2^Department of Otorhinolaryngology, Faculty of Medicine, Cairo University, Giza, Egypt

## Abstract

*Introduction*. Congenital cholesteatoma is a pearly white mass that rarely originates from the mastoid process. *Case Report*. A 21-year-old male patient presented to our department with severe right mastoid pain and postauricular fluctuant swelling for 23 days. There was no preceding history of ear complaints and examination showed a normal right ear drum. Emergency exploration of the mastoid process was done on the same day and revealed localized cholesteatoma limited only to the mastoid cavity. *Conclusion*. Despite a rarity, the mastoid process should be always put in mind as a site of origin for congenital cholesteatoma.

## 1. Introduction

Congenital cholesteatoma (CC) is a pearly white keratinized stratified squamous epithelium that arises in the middle ear cleft. Some diagnostic criteria had been suggested to differentiate it from the acquired cholesteatoma. According to Levenson et al.'s revision, congenital cholesteatoma is a pearly white mass medial to an intact tympanic membrane with normal pars tensa and pars flaccida, with no history of ear discharge or ear drum perforation or any otological procedure [1].

Congenital cholesteatoma may originate from five different sites in the temporal bone: the petrous bone, the cerebellopontine angle, the middle ear cavity, the external ear canal, and the mastoid process. The mastoid process appears to be the least affected and the rarest site that congenital cholesteatoma may arise from [2].

In this study, we present a rare case of mastoid abscess as the only presentation of congenital cholesteatoma in the mastoid process.

## 2. Case Report

A 21-year-old male Yemeni patient presented to the Otorhinolaryngology Department at Sultan Qaboos Hospital in Salalah, Oman, with a history of right mastoid pain of twenty-three- day duration that did not respond to multiple different courses of antibiotics. No preceding history of upper respiratory tract infection was found. The patient did not complain from diminution of hearing or tinnitus or previous history of ear discharge or operations. No history of ear trauma was presented by the patient. The full otorhinolaryngological examination was done. The right ear drum was intact with normal appearance together with the right external auditory canal with no signs of congestion or inflammation. The left ear was normal and tuning fork tests were having within normal results. The right postauricular region showed a tender fluctuant cystic swelling, oval in shape and measuring 2.5 × 3 cm.

The overlying skin was congested red but not attached to the underlying swelling. A defect in the mastoid bone had been felt during the examination. The rest of examination of the ears and the rest of nose and throat examination were normal.

**Figure 1:**
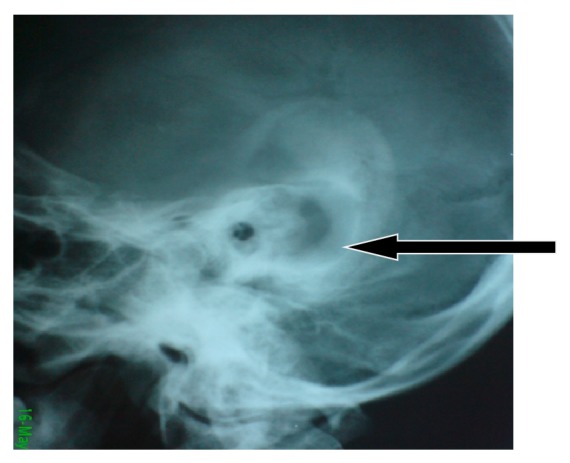
Opacification and large mastoid cavity as seen in the patient's X-ray mastoid.

X-ray mastoid Schuller view was done and revealed opacification of the right mastoid with a picture of large mastoid cavity (Figure 1).

A decision was made for emergency right mastoid exploration to drain the abscess and evaluate the cause.

Informed consent was taken from the patient after explanation of the details of the surgical procedure.

**Figure 2:**
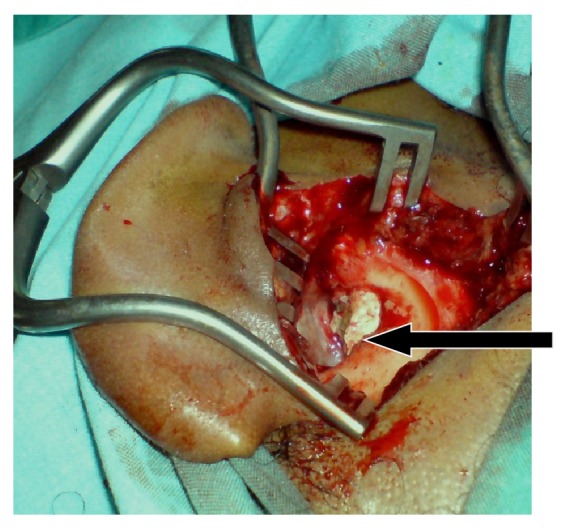
Largely pearly white mass expanding the mastoid cavity.

Under general anesthesia, a right postauricular incision was done and surprisingly a rapid gush of pus appeared after incision of the periosteum. The bony defect was identified and widened. A large pearly white sac with whitish flakes had been seen completely filling the mastoid cavity and widening the mastoid antrum and also encroaching on the facial ridge and reaching posteriorly to the sigmoid sinus and posterosuperiorly to the sinodural angle (Figure 2).

**Figure 3:**
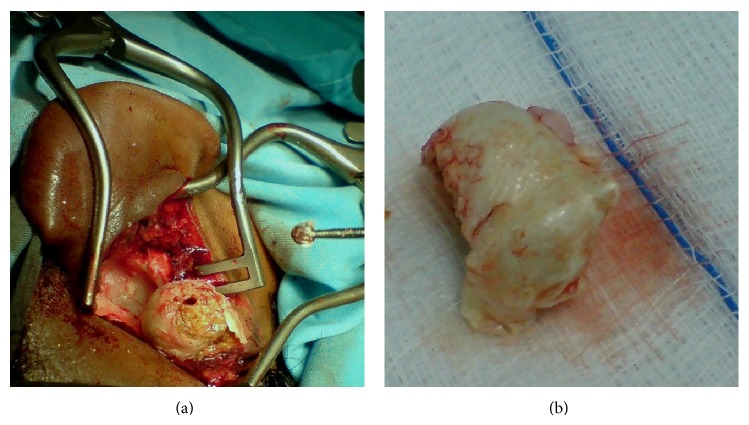
(a) shows the sac after delivery from the mastoid cavity before its removal. (b) shows the sac.

The sac was completely delivered in total (Figure 3), and the wound was closed after insertion of a drain in the large cavity left after removal of the sac.

The specimen was sent for histopathology that confirmed the diagnosis of cholesteatoma. The patient had no complications intraoperatively or in the postoperative period. Stitches and ear pack had been removed ten days after the operation and the patient had been followed up for three months later on with clean wound site and no recurrence of the swelling.

## 3. Discussion

Different studies and theories have been suggested to explain the origin of congenital cholesteatoma as metaplasia theory [3], invagination theory [4], epithelial rest theory [5], and implantation theory [6].

It is somehow difficult to apply the metaplasia theory in our case in this paper as it is strange that metaplasia may affect the mastoid process leaving a completely normal middle ear cavity. This is supported by Friedberg's study in 1994 [6].

The invagination theory of the ectoderm can be applied; however, it is extremely rare. Also, the epithelial rest theory can be applied if epidermoid formation happens to the underdeveloped mastoid during the fetal life and grows later into congenital cholesteatoma yet this theory is also rare [7].

The implantation theory might be accepted in our case. Canalis et al., in 2002, suggested that cranial cholesteatoma can arise with entrapment of squamous epithelium in the suture during the period of mastoid fontanelle closure leading to the formation of congenital cholesteatoma in the mastoid process [8].

The most accepted theory for the development of congenital cholesteatoma is the epithelial cell rests. It depends on Teed's initial observation of an epidermal structure in a 5-month human fetus in “the dorsal lateral pole of the tympanum, just medial to the neck of the malleus” [9].

These rests are ectodermal implants in the fusion plates between the first and second branchial arches that appear around 10 weeks at the junction of the first branchial cleft and pouch systems [10].

Levenson et al. [1] postulated that these rests helped in the middle ear and tympanic membrane development and that they are initially dormant. These epithelial cell rests undergo rapid proliferation before resorption around 33 weeks of gestation. In cases where incomplete resorption is the case, it is thought that congenital cholesteatoma will form. Levenson et al. [1] postulated that the epidermal rests fail to undergo involution because of continuous and chronic irritation.

Michaels [11] confirmed these rests of epithelial cells histologically in 54% of the fetal temporal bones examined and postulated that their persistence was the cause of congenital cholesteatoma.

Mastoid abscess was the first presentation of congenital cholesteatoma in a 21-year-old male patient in our study. Migirov et al. presented seven cases with mastoid subperiosteal abscess as the first presentation of congenital cholesteatoma in the pediatric population, of whom two patients presented with normal intact tympanic membrane. All the seven patients had no history of middle ear disease [12].

Hidaka et al., in 2010, reported an adult case with acute mastoiditis as the first presentation of congenital cholesteatoma with no extension in the attic or aditus ad antrum as seen in the operation and suggested that any adult with mastoiditis should be evaluated for congenital cholesteatoma. They also mentioned that, including their case, only four cases till that time presented with mastoid pain or swelling [13]. Another presentation of mastoid congenital cholesteatoma mentioned in the literature was a stricture in the external auditory canal with intact tympanic membrane [14].

According to Lee et al. and Hong et al.'s reviews in 2007 and 2014, about 30 cases of mastoid congenital cholesteatoma existed [7, 15] to which we add the case presented in this study.
